# Retraction Note: Exosomal lncRNA HNF1A-AS1 affects cisplatin resistance in cervical cancer cells through regulating microRNA-34b/TUFT1 axis

**DOI:** 10.1186/s12935-024-03217-4

**Published:** 2024-01-18

**Authors:** Xiaoqiong Luo, Jingxi Wei, Feng-lian Yang, Xiao-xia Pang, Feng Shi, Yu-xia Wei, Bi-yun Liao, Jun-li Wang

**Affiliations:** 1 Center of Reproductive Medicine, Affiliated Hospital of Youjiang Medical College for Nationalities, Zhongshan Second Road 18th, Baise, 533000 Guangxi China; 2 Youjiang Medical College for Nationalities, Baise, 533000 People’s Republic of China

**Retraction: Cancer Cell Int (2019) 19:323** 10.1186/s12935-019-1042-4

The Editors-in-Chief have retracted this article. Concerns were raised regarding a number of figures, specifically:

- Figure 1e: appears to be overlapped between the panels for HeLa/S sh-NC and HeLa/DDP sh-NC.
- Figure 2a appears to contain multiple instances of repeating features.
- Figure 2b appears to be also published as Fig. 2B in an article by different authors that was simultaneously under consideration at a different journal [1].
- Figure 5c appears to contain data points that were added at different stages.
- Figure 6c: the original background for the tumors appears to have been removed.

The Editors-in-Chief therefore no longer have confidence in the results and conclusions of this article.

Corresponding author Jun-li Wang has stated that all authors agree with this retraction.
